# Phacolytic glaucoma – case report

**DOI:** 10.22336/rjo.2021.38

**Published:** 2021

**Authors:** Mioara Laura Macovei, Mădălina Canache, Bianca-Mihaela Neagoe

**Affiliations:** * Ophthalmology Department, “Dr. Carol Davila” Central Military Emergency University Hospital, Bucharest, Romania

**Keywords:** lens-induced glaucoma, phacolytic glaucoma, cataract

## Abstract

We presented a case of a 76-year-old male patient with phacolytic glaucoma and a rather atypical clinical presentation. The sudden onset of intense pain forced the patient to seek medical help and by doing so, further damage of the optic nerve was prevented and a good visual acuity was obtained following cataract surgery.

## Introduction

Lens induced glaucoma (LIG) is a secondary glaucoma in which the lens plays an essential pathogenic role, either due to an increase in its thickness, change in its position or by an inflammatory process [**1**]. Phacomorphic glaucoma accounts for most cases of LIG followed by phacolytic glaucoma [**2**]. Phacolytic glaucoma, a rare secondary open-angle glaucoma, is caused by leakage of high molecular weight proteins through the capsule of a hypermature cataract [**3**]. The clinical presentation usually consists of a painful eye, decreased vision and conjunctival hyperemia. 

## Case report

A 76-year-old male patient presented with intense pain, redness, and visual acuity loss in his left eye (LE). The symptoms had a sudden onset the night before, and were followed by headache, nausea, and vomiting. He denied any recent trauma. He had undergone cataract removal with intraocular lens (IOL) implantation in his right eye (RE) in 2007, and experienced a gradually blurring of his left eye visual field ever since. The family history was not relevant, he had no known allergies and the past medical history consisted of hypertension, ischemic cardiomyopathy with an old myocardial infarction, congestive heart failure, type II diabetes, obesity, and benign prostatic hyperplasia.

The patient had a best-corrected visual acuity (Snellen chart) of 20/ 25 in the right eye and light perception with good projection in the left eye. The intraocular pressure (IOP) was measured using non-contact tonometry (NCT) for his right eye (RE: 20 mmHgNCT), and Goldmann applanation tonometry (GAT) for his left eye (LE: 48 mmHgGAT). Both eyes had normal ocular motility. 

**Fig. 1:**
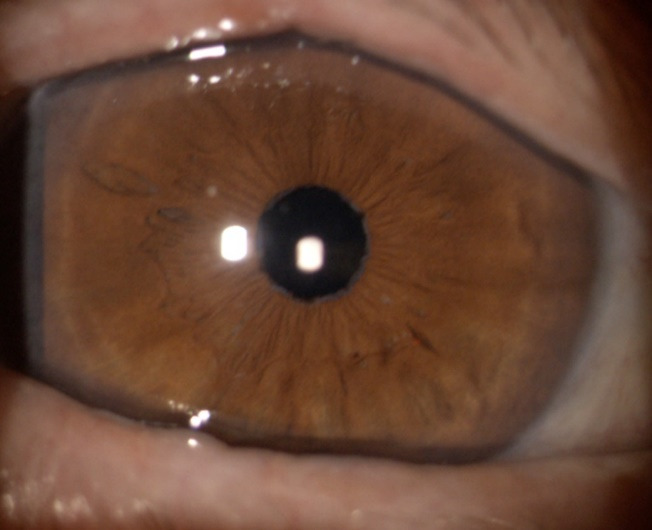
Slit lamp photo - RE: normal anterior ocular segment, pseudophakic eye

Slit lamp examination of the RE showed: clear cornea, deep anterior chamber with transparent aqueous humour, normal iris architecture, normal pupillary reflexes and an intracapsular IOL (**Fig. 1**).

**Fig. 2:**
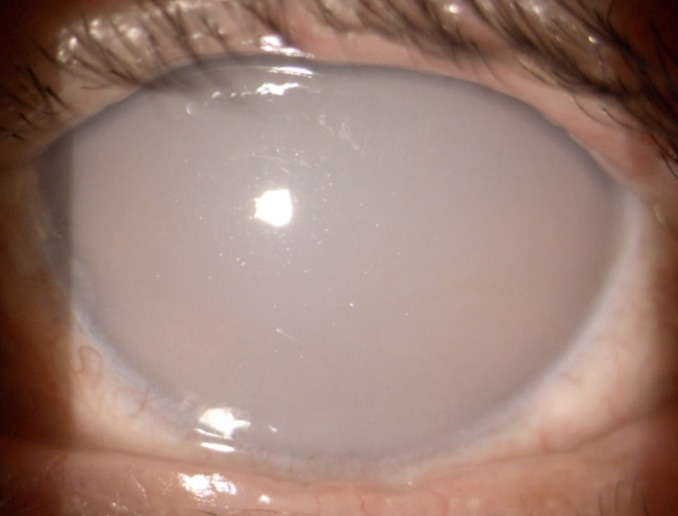
Slit lamp photo - LE: milky appearance of the anterior chamber

The LE had mild conjunctival injection and corneal oedema, the endothelium was covered with shiny white crystals and the aqueous humour had a milky appearance that obstructed the visualization of further ocular structures (**Fig. 2**).

Dilated fundus examination of the RE revealed a normal optic disc, cup to ratio 0.3, pigmentary changes in the macula, angiosclerosis and a normal peripheral retina. Fundoscopic examination of the LE was not possible. 

**Fig. 3:**
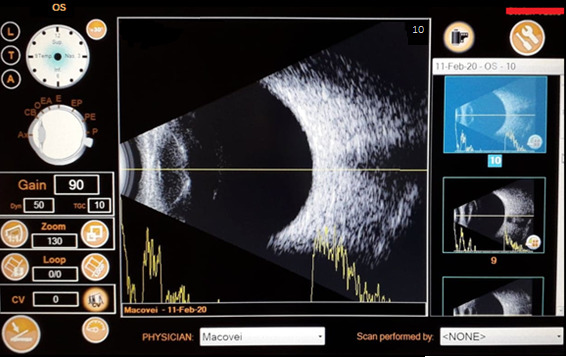
Ultrasound imaging LE: axial scan

A simultaneous A and B-mode ultrasonography of the left eye showed a highly reflective posterior lens surface displaced towards the anterior vitreous. There was no retinal detachment and no mass lesion (**Fig. 3**).

The patient was diagnosed with a pseudophakic right eye and considering no history of previous ocular trauma, phacolytic glaucoma was suspected for the left eye. Lens-particle glaucoma was the primary differential diagnosis. Endogenous endophthalmitis and Masquerade syndrome were unlikely and were soon excluded based on normal clinical and paraclinical evaluation.

Considering the rise of the IOP, the patient received pressure lowering topical and systemic therapy. Topical dorzolamide/ timolol and brimonidine were given to the patient twice daily and additionally he received oral acetazolamide 250 mg and magnesium/ potassium aspartate 2 times daily. Fluorometholone 0.1% 3 times daily was used as topical anti-inflammatory agent. The IOP normalized under treatment and the patient was scheduled for surgery the next day. 

Combining the data obtained by optical biometry and by A-scan applanation ultrasonography for the left eye, with the axial length measurement for the pseudophakic contralateral eye, a 20 diopters monofocal IOL was selected for intraocular implantation. 

Prior to surgery, local anaesthesia was achieved by retrobulbar block. Intraoperative, a Morganian cataract with an intact capsule was visualized following the anterior chamber (AC) washout, thus, the diagnosis of phacolytic glaucoma was confirmed. After attaining an adequate mydriasis by intracameral injection of 1 mL epinephrine hydrochloride 0.001%, the AC was deepened using a dispersive ophthalmic viscoelastic device (OVD) and a successful continuous curvilinear capsulorhexis was created. Hydrodissection and hydrodelineation were not necessary as the nucleus was already mobile within the capsule. After phacoemulsification, white cortical lens matter was seen into the anterior vitreous and localized anterior vitrectomy was performed through a small breach made into the posterior capsule. The capsular bag was filled with a cohesive OVD and the IOL was placed into-the-bag. After removing the OVD from the capsular bag and anterior chamber, the corneal incisions were hydrated with basic saline solution and a 10.0 nylon suture was placed at the main corneal incision for a better wound closure. A subconjunctival dexamethasone-gentamicin injection was given at the completion of the cataract surgery.

**Fig. 4:**
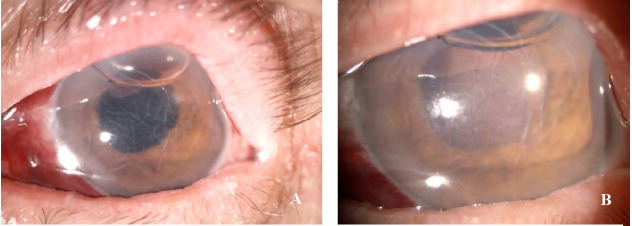
**A** Post-operative day 1, pharmacological mydriasis. Slit lamp photo: Descemet folds, wide AC with clear aqueous humour, IOL in proper position; **B.** Post-operative day 1. Slit lamp photo with additional magnification showing the red reflex

On the first postoperative day, the patient had a mild subconjunctival haemorrhage, corneal haze associated with mild stromal oedema, Descemet’s folds, normal anterior chamber depth with clear aqueous humour, normal pupillary reflexes, and the IOL was well positioned into the capsular bag (**Fig. 4A**). The corneal suture was in situ with minimal discomfort, the IOP was 17 mmHgGOT and the patient had a visual acuity of 20/ 200. The red reflex was present, but fundus details could not be clearly observed (**Fig. 4B**). 

The patient was discharged with clear postoperative instructions and restrictions. An antibiotic was prescribed to protect against endophthalmitis, a steroid to control inflammation and a nonsteroidal anti-inflammatory drug to complement the steroid in controlling inflammation and to prevent the developing of cystoid macular oedema. A hypertonic sodium chloride (5%) eye drop was recommended in order to relieve corneal oedema. The cataract removal normalized the IOP so that ocular hypotensive drugs were no longer required.

**Fig. 5:**
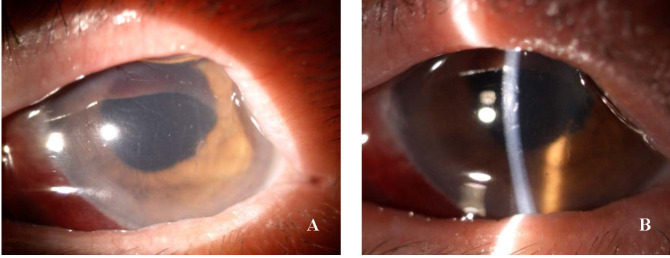
**A** Post-operative day 7, pharmacological mydriasis. Slit lamp photo: Descemet folds, wide AC with clear aqueous humour, IOL in proper position; **B.** Slit lamp photo, narrow light beam: Descemet folds, wide AC

By the 7th post-operative day, the conjunctival haemorrhage was partially resorbed, the corneal oedema was significantly reduced (**Fig. 5A**, **B**), and fundus examination was possible, revealing a normal optic disc, cup to ratio 0.3, pigmentary changes in the macula, angiosclerosis and a normal peripheral retina. The IOP was 18 mmHgNCT and the vision improved to 20/ 80. The corneal suture was removed under topical anaesthesia. 

**Fig. 6:**
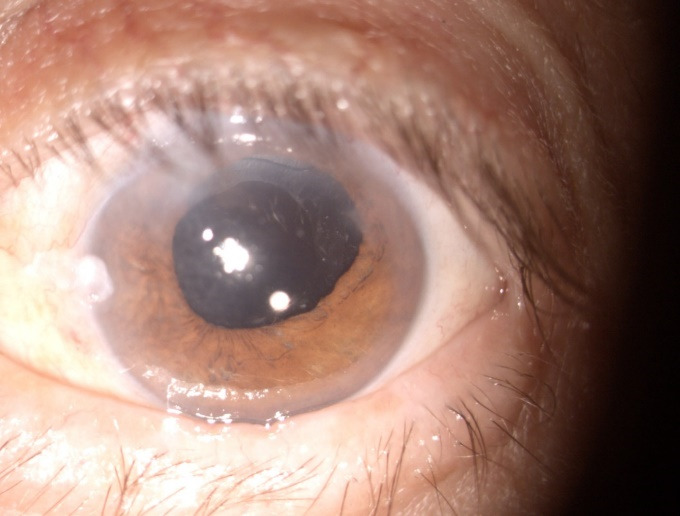
Post-operative day 21. Slit lamp photo: clear cornea, pharmacologically induced mydriasis, into-the-bag IOL

The evolution improved gradually under treatment. The 3-weeks examination revealed a transparent conjunctiva and a clear cornea, the IOL was stable in the capsular bag no additional fundus abnormalities being observed (**Fig. 6**). The visual acuity was 20/ 32 and the IOP was still within the normal range (19 mmHgNCT).

## Discussion

Lens-induced glaucoma (LIG) is an important cause of secondary glaucoma in the developing world [**4**] and comprises several different glaucomatous processes that share the role of the lens in their pathogenesis [**5**]. LIG can be divided into two major categories, presenting as either secondary angle-closure or secondary open-angle glaucoma. The first category relates to a blockage of the aqueous humour flow from the posterior to the anterior chamber and can be caused by lens swelling (phacomorphic glaucoma) or lens dislocation (ectopia lentis). The second category is characterized by trabecular meshwork blockage from lens proteins (phacolytic glaucoma), liberated fragments of lens material after capsular disruption following cataract extraction, lens trauma, or Nd:YAG posterior capsulotomy (lens particle glaucoma) or from an inflammatory reaction directed against own lenticular antigens (phacoantigenic glaucoma) [**5**]. 

Phacomorphic glaucoma is the main cause of lens-induced glaucoma, followed by phacolytic glaucoma [**2**,**6**,**7**]. This latter condition is rare in developed countries with adequate access to ophthalmologic care and more common in under-developed countries. One study from Nepal diagnosed phacolytic glaucoma in 0.4% of presenting cataracts [**2**,**8**].

Phacolytic glaucoma occurs primarily in the setting of a senile hypermature, Morgagnian, cataract [**9**]. Over time, the cortical lens fibers degenerate into hydrosoluble protein aggregates. These high-molecular-weight lens proteins leak through a grossly intact lens capsule into the anterior chamber, where they induce macrophage activity [**8**,**10**]. A key defining feature of phacolytic glaucoma has been the presence of an intact lens capsule. However, a 2014 study that used electron microscopy to evaluate the capsule of a patient found multiple full thickness dehiscences and holes despite an intact appearance both macroscopically and histologically [**8**,**11**]. The combination of protein-laden macrophages and high-molecular-weight proteins in the anterior chamber leads to trabecular meshwork obstruction resulting in intraocular pressure elevation and secondary open-angle glaucoma [**9**].

Multiple studies showed females outnumbered males in both phacomorphic and phacolytic glaucoma [**5**,**12**,**13**].

Typically, the clinical presentation is that of an elderly patient who complains of an acute onset of severe pain, redness, and worsening of vision [**8**]; he has a history of a gradual decrease of visual acuity over the preceding months or years, reflecting the slow maturation of the cataract. On examination, the IOP is very high, accounting for the resented pain [**9**]. 

Slit lamp biomicroscopy usually shows microcystic corneal oedema, a hypermature cataract and a deep anterior chamber. The cellular reaction in the AC is variable, from mild cells and flare to an intense reaction [**9**]. Large floating white particles, consisting of lens protein and protein-containing macrophages may impart a milky appearance to the aqueous if very dense and can form a pseudohypopyon. If a reasonable view can be obtained, gonioscopy shows an open angle with lens-derived material and inflammatory cells that are most substantial inferiorly [**14**].

Although the diagnosis is based upon clinical features, microscopic examination of aspirated aqueous humour can aid in suspected cases. Biochemical studies can help identify high-molecular-weight lens proteins that have leaked out of the cataractous lens [**9**].

Phacolytic glaucoma is typically handled as an emergency. After the IOP is controlled medically with topical aqueous suppressants (beta-blockers, alpha-2 agonists, and carbonic anhydrase inhibitors) and, if needed, with systemic carbonic anhydrase inhibitors and osmotic agents, the proteinaceous material is washed out from the anterior chamber and the cataract is removed [**9**]. 

Though curable, cataract remains the most important cause of blindness in developing countries, mostly affecting the elderly. Delayed presentation for treatment ends up in serious complications such as lens-induced glaucoma, which may cause irreversible sight loss. Cataract surgery is the most effective treatment for lowering the IOP and visual recovery in these patients. In a prospective observational study conducted by Calcutta National Medical College in 2012-2013, most of the patients (72,2%) had a postoperative best corrected visual acuity of more than 20/ 125 after complete follow-up [**15**]. In this case report, the sudden onset of intense pain forced the patient to seek medical help and by doing so, further damage of the optic nerve was prevented and a good visual acuity was obtained following cataract surgery. 

## Conclusions

Phacolytic glaucoma is a complication of a hypermature cataract and typically occurs in elderly patients.

The prognosis for a good visual recovery depends on an early diagnosis and management that aids IOP control and prevents optic nerve damage. 

**Conflict of Interest statement**

The authors state no conflict of interest.

**Informed Consent and Human and Animal Rights statement**

Informed consent has been obtained from all individuals included in this study.

**Authorization for the use of human subjects**

Ethical approval: The research related to human use complies with all the relevant national regulations, institutional policies, is in accordance with the tenets of the Helsinki Declaration, and has been approved by the institutional review board of “Dr. Carol Davila” Central Military Emergency University Hospital, Bucharest, Romania.

**Acknowledgments**

None.

**Sources of Funding**

None.

**Disclosures**

None.
